# Production of Chicken Patties Supplemented with Cantaloupe By-Products: Impact on the Quality, Storage Stability, and Antioxidant Activity

**DOI:** 10.1155/2022/9918215

**Published:** 2022-03-14

**Authors:** Maha IK Ali, Rehab Mohamed Ibrahim, Aliaa G. M. Mostafa

**Affiliations:** ^1^Department of Special Food and Nutrition Research, Food Technology Research Institute, Agricultural Research Center, Giza, Egypt; ^2^Department of Meat and Fish Technology Research, Food Technology Research Institute, Agricultural Research Center, Giza, Egypt

## Abstract

This study investigated the effect of supplementation with cantaloupe peel (CP) and seeds (CS) (3, 6, 9, and 12%) powder on the quality and antioxidant activity of raw and cooked chicken patties during storage (-20°C/3 months). The addition of CP and CS powder increased protein, fat, ash, and fiber values of chicken patties compared with control, while carbohydrate, pH, and TBA were decreased at zero time and after 3 months of storage. The WHC, cooking yield, fat retention, and moisture retention were increased by increasing CP and CS powder addition ratios, while cooking loss and shrinkage were decreased. Also, CP and CS powder improved antioxidant activity, microbiological quality, and overall acceptability of chicken patties. The hardness of raw and cooked chicken patties was decreased with increasing CP and CS addition ratios. It is recommended to use CP and CS powder as functional ingredients in the preparation of functional foods.

## 1. Introduction

The by-products of fruits such as peels, seeds, and unused flesh are usually wasted and disposed of its. These agro-industrial residue wastes are a serious problem that must be managed effectively, and the fruits are natural sources of active compounds like vitamins, total phenolic, dietary fibers, and carotenoids [1, 2].

In recent years, research has focused on peels and seeds of fruits due to their important nutritional and medicinal properties. In addition, the vegetable oil extracted from seeds has an excellent source of biologically active compounds and antioxidants [3, 4].

Cantaloupe melon (*Cucumis melo* L.) is one of the most consumed crops all over the world because of its sweetness and delicious flavor; it belongs to *Cucurbitaceae*, a family which includes a large group of species with great economic importance. Often, cantaloupe peel and seeds that are agro-industrial residue waste products are eliminated and are a rich source of phytochemicals such as polyphenol compounds, carotenoids, and other substances that have a positive effect on health. Also, these polyphenols have antioxidant activity as well as work to prevent the oxidation of fats and protect cells from damage by reactive oxygen species (ROS) [5, 6].

Cantaloupe peel is one of the by-products that are eliminated. Several studies have indicated that the peel is rich in phenolic compounds, flavonoids, carotenoids, and other bioactive components that have a positive effect on health [7]. Furthermore, Goulas and Manganaris [8] reported that the peel contains had higher phenolic compounds and vitamin C contents than the pulp. Also, Mallek-Ayadi et al. [1] reported that the cantaloupe peel contained carbohydrates (69.77%), ash (3.67%), total dietary fibers (41.69%), and antioxidants like polyphenols and flavonoids (332 mg/100 g extract and 95.46 mg/100 g extract, respectively). In addition, it is considered as a good source of minerals, such as calcium, potassium, and magnesium.

*Cucumis melo* L. seeds are an excellent sources of protein, lipids, and fiber. Also, they are rich sources of minerals especially magnesium, phosphorus, sodium, and potassium. In Arab countries, the seeds are used after salting and roasting as a snack, as well as being dried and used as a flavoring agent for Indian dishes and sweets [9, 10]. It is also a rich source of antioxidants and is also used to maintain shelf life [11]. In addition, Mansouri et al. [12] reported that the seed kernel of *Cucumis melo* is rich in unsaturated fatty acids like linoleic and linolenic as it contains 40-50% of the fatty acids and 20%-30% of the protein; it is considered as a good source of essential amino acids, such as isoleucine, methionine, tyrosine, phenylalanine, and valine [13–15]s, and this depends on the variety of *Cucumis melo*. As well, it were found that fatty acids ranged in oil from 41.6% to 44.5%. Also, it is a good source of fiber, minerals, and biologically active compounds [16, 17]. It is a new food ingredient. Currently, it is used in some foods such as bakery products [18].

Nowadays, the meat industry is interested in replacing with synthetic antioxidants such as BHT, BHA, and TBHQ, etc which are toxic and harmful to health with natural products of plant origin that contain biologically active compounds with their health benefits [19, 20]. And scientific evidence confirmed that the use of natural antioxidants would reduce fat oxidation [21], especially in chicken products most prone to deterioration.

Poultry meat is one of the most consumed meats in the world due to its availability and cheap price. It is an excellent source of biological protein, minerals, and vitamins and has low fat content, in addition to being rich in unsaturated fatty acids that can lead to oxidation, which leads to a decrease in meat quality and therefore low acceptance to its consumer [22].

Lipid oxidation and color are the most important factors that attract consumers to accept meat and its products; several studies have noted a decrease lipid oxidation in different meats, such as pork, beef, lamb, chicken, and goat after adding oregano, sage, rosemary, thyme, marjoram, caraway, basil, ginger, kinbow, pomegranate, cereal, walnut, and seaweed [19, 23–26]. It is worth to mention that a diet containing meat is rich in energy, saturated fatty acids, and cholesterol and poor in dietary fiber. The diet must contain fiber and incorporate it into food products to take advantage of important functional properties and technology [27–29]. Therefore, the aim of this study is to investigate the effect of supplementation of chicken patties by cantaloupe peel and seeds powder on the quality, storage stability, functional properties, antioxidant activity, and overall acceptability.

## 2. Materials and Methods

### 2.1. Materials

Fresh cantaloupe (*Cucumis melo* L.) fruit and fresh chicken breast meat, eggs, bread crumbs, salt, spices mixture (ground black pepper, ground cumin, and onion powder), and corn oil were obtained from a local market in Alexandria, Egypt. Grease-proof paper, polyethylene bags, and foam plates (22 × 17 cm) were purchased from Alexandria Local Market, Egypt.

All reagents and chemicals used in this study were of analytical grade. 1,1-diphenyl-2-picryl-hydrazyl (DPPH) was purchased from Sigma-Aldrich (Munich, Germany). Ferric chloride, potassium ferricyanide, and gallic acid were obtained from Loba Chemie, Mumbai, India.

### 2.2. Methods

#### 2.2.1. Technological Methods

*(1) Preparation of Cantaloupe Peel and Seeds Powder*. Cantaloupe fruit was washed thoroughly with tap water; then, peel and seeds were separated from the pulp fruit using a knife. The peels were shredded into small pieces. Peels and seeds were dried in a hot air oven dryer at 45°C for approximately 16-18 hours till its moisture content reached 8%, then ground into a fine powder with an electric mill (Moulinex, MC3001), then packed into polyethylene bags, and kept at -20°C until used.

*(2) Preparation of Chicken Patties*. Chicken patties were prepared according to the method described by Nardoia et al. [30] with some modifications, by mixing of minced chicken meat, whole egg, bread crumbs, spices mixture, and salt (Table 1). The bread crumbs in the formulas were replaced with 3, 6, 9, and 12% of cantaloupe peel (CP) or seeds (CS) powder. The chicken patties formulas were homogenized in a Braun Cutter Machine (Combi Max 700, USA) and then formed and processed into chicken patties (50 g weight, 10 cm diameter, and 1 cm thickness). A plastic packaging film was used to help maintain the shape of the chicken patties and kept at –20°C for 3 months. The samples were analyzed on zero time and after 3 months of frozen storage.

### 2.3. Cooking Procedure of Chicken Patties

Chicken patties samples were grilled in a non-sticky pan (electric pan) with no added fat for 5 min on one side and 3 min for the other side, then cooled to room temperature (22 ± 3°C) as described by Mohamed and Mansour [31].

### 2.4. Analytical Analysis

#### 2.4.1. Proximate Chemical Composition

Proximate chemical composition including moisture, crude protein, crude fat, total dietary fiber, and total ash was determined in triplicate according to the procedures of AOAC [32]. Total carbohydrates were calculated by difference. Total caloric values (Kcal) were calculated using the following equations according to Ali et al. [33]:

(1) $$\begin{array}{c} \text{Total calories}=4\left(\text{protein}+\text{carbohydrates}\right)+9\left(\text{fat}\right). \end{array}$$

#### 2.4.2. Determination of Minerals

Minerals including calcium (Ca), potassium (K), magnesium (Mg), iron (Fe), zinc (Zn), and sodium (Na) were measured in ash solution using ICP-OES Agilent 5100 VDV according to U.S.EPA [34].

#### 2.4.3. pH Values

10 g of each sample was blended with 100 mL distilled water for 60 s in a homogenizer. The pH values were measured in homogenate samples using a pH meter (Martini, Italy) according to Naveena et al. [26].

#### 2.4.4. Thiobarbituric Acid (TBA) Assay

The thiobarbituric acid (TBA) was calorimetrically estimated according to Park et al. [35] using a UV-VIS spectrophotometer (Laxco Alpha 1102) expressed as milligrams of malonaldehyde per kilogram.

### 2.5. Functional Properties of Peel and Seeds

#### 2.5.1. Water Retention Capacity

The water retention capacity (WRC) was measured following the method of Garau et al. [36]. Ground samples of melon peels (0.5 g) were hydrated in excess during 24 h in a 50 mL tube, prior to centrifugation at 2000 × g for 25 min. Excess supernatant was decanted. Water retention was recorded in terms of grams of water per gram of dry sample.

#### 2.5.2. Oil Retention Capacity

The oil retention capacity (ORC) was conducted according to Garau et al. [36] method. Ground samples of melon peels (0.5 g) were mixed with sunflower oil (10 mL) and centrifuged at 2000 × g for 20 min, and the excess supernatant was decanted. Oil retention capacity was expressed as grams of oil per gram of dry sample.

### 2.6. Functional Properties of Chicken Patties

#### 2.6.1. Fat Retention Value

The fat retention value represents the amount of fat retained in the product after cooking, and it was calculated according to Murphy et al. [37] by using the equation as follows:

(2) $$\begin{array}{c} \text{Fat retention }\left(\%\right)=\frac{\text{ cooked weight }\left(\text{g}\right)\times \text{fat in cooked }\left(\%\right)}{\text{uncooked weight }\left(\text{g}\right)\times \text{fat in uncooked }\left(\%\right)}*100. \end{array}$$

#### 2.6.2. Moisture Retention

Moisture retention was determined according to Aleson-Carbonell et al. [38]:

(3) $$\begin{array}{c} \text{Moisture retention}\left(\%\right)=\frac{\text{ cooked weight }\left(\text{g}\right)\times \text{moisture in cooked samples }}{\text{raw weight }\left(\text{g}\right)\times \text{moisture in raw samples}}*100. \end{array}$$

### 2.7. Cooking Measurement of Chicken Patties

#### 2.7.1. Water Holding Capacity (WHC)

Water holding capacity (WHC) and plasticity (cm^2^/0.3 g) were determined by filter press method as described by Wang and Zayas [39].

#### 2.7.2. Cooking Loss (%)

The cooking loss was calculated according to Jama et al. [40].

(4) $$\begin{array}{c} \text{Cooking loss }\left(\%\right)=\frac{\text{weight of raw sample }\left(\text{g}\right)–\text{weight of cooked sample }\left(\text{g}\right)}{\text{weight of raw sample }\left(\text{g}\right)}*100. \end{array}$$

#### 2.7.3. Cooking Yield (%)

The cooking yield was calculated according to Aleson-Carbonell et al. [38].

(5) $$\begin{array}{c} \text{Yield }\left(\%\right)=\frac{\text{weight of cooked sample }\left(\text{g}\right)}{\text{weight of raw sample}\left(\text{g}\right)}*100. \end{array}$$

#### 2.7.4. Change of Chicken Patties Thickness (%)

The change in chicken patties thickness (measurements were taken using calibers) was calculated according to Serdaroğlu et al. [41].

(6) $$\begin{array}{c} \text{The change in thickness}=\frac{\text{uncooked thickness}–\text{cooked thickness}}{\text{uncooked thickness}}*100. \end{array}$$

#### 2.7.5. Change of Chicken Patties Shrinkage (%)

Change in shrinkage for prepared chicken patties samples was measured before and after sample cooking according to George and Berry [42] using the following equations:

(7) $$\begin{array}{c} \text{Change in shrinkage }\left(\%\right)=\frac{\left(\text{uncooked diameter }\left(\text{cm}\right)–\text{cooked diameter }\left(\text{cm}\right)\right)}{\text{uncooked diameter }\left(\text{cm}\right)}*100. \end{array}$$

### 2.8. Determination of Antioxidant Activity

Antioxidant activity was determined during a storage period of three months using three assays. All determinations were carried out in triplicate.

#### 2.8.1. Total Phenolic Contents

Total phenolic contents (TPC) were determined in triplicate using the method developed by Abirami et al. [43]. One and a half milliliters of Folin–Ciocalteu's reagent (diluted 10 times) and 1.2 mL of Na_2_CO_3_ (7.5% *w*/*v*) were added to 300 *μ*L of water-soluble extract. Mixtures were shaken and kept at room temperature for 30 min before measuring absorbance at 765 nm using a spectrophotometer (Pg T80+, England). TPC was expressed as gallic acid equivalent in milligrams per milliliter of extract.

#### 2.8.2. DPPH Scavenging Activity (%)

Radical scavenging activity of samples was measured using DPPH (1,1-diphenyl-2-picrylhydrazyl) according to Brandwilliams et al. [44]. The percentage of DPPH scavenging activity (%) for samples was calculated using the equation as follows:

(8) $$\begin{array}{c} \text{DPPH scavenging activity }\left(\%\right)=\frac{\text{Abs control}-\text{Abs sample}}{\text{Abs control}}*100. \end{array}$$

#### 2.8.3. Ferric Reducing Antioxidant Power (FRAP)

Ferric reducing antioxidant power was determined according to Gutteridge and Halliwell [45]. One milliliter of extract was mixed with 2.5 mL of phosphate buffer (0.2 M, pH 6.6) and 2.5 mL of potassium ferricyanide (1% *w*/*v*), and then, the mixture was incubated at 50°C for 30 min. After incubation, 2.5 mL of TCA (10% *w*/*v*) was added and the mixture was centrifuged at 1650 rpm/10 min. Finally, 2.5 mL of the supernatant solution was mixed with 2.5 mL of distilled water and 0.5 mL of FeCl_3_ (0.1% *w*/*v*) and the absorbance was measured at 700 nm using a spectrophotometer (Pharmacia, USA). The FRAP values, expressed in milligrams of ascorbic acid per 100 mL, were derived from a standard curve.

### 2.9. Microbiological Evaluation

Total plate count (TPC) was determined for the samples at zero time and after 3 months of frozen storage (-20°C) by using pour plate method and plate count agar as medium according to ISO 8443 [46]. For coliform group bacteria, pour plate procedure and Violet Red Bile Agar medium were used according to ISO 4832 [47]. Regarding yeasts and molds, they were determined by plating 0.05 mL of diluted sample on potato dextrose agar (Oxoid CM) and incubated for 5 days at 25°C; yeast and mold colonies were counted separately according to ICMSF [48].

### 2.10. Color Measurement

The color values, which includes lightness (*L*∗), redness (*a*∗), and yellowness (*b*∗) of chicken patties samples, were evaluated using a HunterLab UltraScan VIS model colorimeter (USA), as described by Santipanichwing and Suphantharika [49].

### 2.11. Texture Profile Analysis

Texture profile analysis of different chicken patties samples were determined by a universal testing machine (Cometech, B type, Taiwan) provided with software. An aluminum 25 mm diameter cylindrical probe was used in a “Texture Profile Analysis” CT V1.2 Build 9 (TPA) double compression test to penetrate to 50% depth, at 2 mm/s speed test. Hardness (g/s), springiness (mm), cohesiveness (ratio), gumminess (g/s), chewiness (mJ), and resilience were calculated from the TPA graphic.

### 2.12. Sensory Evaluation

Color, taste, odor, texture, and overall acceptability of cooked chicken patties were evaluated using 10 trained panelists from Food Technology Research Institute Agricultural Research Center, Alexandria. A 9-point hedonic scale was used (9 = like extremely and 1 = dislike extremely) according to Meilgaard [50].

### 2.13. Statistical Analysis

The statistical analysis was performed using one-way analysis of variance (ANOVA) using SAS statistical analysis software [51]. Means were compared by Duncan's test at the significance level of *p* < 0.05.

## 3. Results and Discussion

### 3.1. Proximate Composition and Mineral Content of Cantaloupe Peel and Seeds Powder

Proximate composition and mineral content of cantaloupe peel and seeds powder which was used to prepare chicken patties samples are presented in Table 2. Results showed that the cantaloupe peel powder (CP) was significantly higher in moisture, total carbohydrate, and total dietary fiber (17.99%, 68.80%, and 39.33%, respectively), than seeds powder (CS) (7.20%, 31.10%, and 24.17%, respectively). Furthermore, CP had lower values in fat, ash, and protein. These results are in agreement with those found by Mallek-Ayadi et al. [1], the moisture, crude protein, ash, crude fiber, fat, and total carbohydrate in cantaloupe peel were 16.95%, 7.48%, 2.93%, 3.67%, 41.69%, 2.12%, and 69.77%, respectively. Also, da Cunha et al. [52] found that the cantaloupe seeds flour contains 2.64% moisture, 17.64% crude protein, 4.12% ash, 35.48% total dietary fiber, and 30.43% fat. Also, results revealed that CP had the highest content in some minerals such as calcium (1000 mg/100 g) and potassium (940 mg/100 g). On the other side, the CS contains high amounts of magnesium, iron, zinc, and sodium (900, 2.50, 2.10, and 289 mg/100 g), respectively. The data obtained in the present study are similar mostly with those reported by Mallek-Ayadi et al. [1] and Mallek-Ayadi et al. [53].

Data in Table 2 showed that the higher water retention capacity (5.96 g/H_2_O) was observed with CP compared to CS (4.43 g/H_2_O). This might be due to the higher fiber content of CP. Results cleared that the oil retention capacity of peel powder is similar to that of seeds powder. These results are in line with those reported by da Cunha et al. [52]. Currently, the property of fat retention capacity was exploited in foods, especially meat products, which are lost during the cooking process, and this is likely to be useful to improve flavor and yield [54].

### 3.2. Antioxidant Activity of Cantaloupe Peel and Seeds Powder

In terms of the antioxidant activity of CP and CS (Table 2), results revealed that CP was significantly (*p* < 0.05) higher in total phenolic (1050 GAE mg/100 g), DPPH (91.34%), and FRAP value (700 mg AAE/100 g). These results are less than those reported by Vella et al. [55] who reported that the total polyphenol content of cantaloupe peel and seeds were 25.48 and 1.50 mg GAE/g while FRAP values were 12.27 and 0.31 mg AAE/g, respectively. This may be due to the difference in the variety, degree of maturity, and environmental factors like geographic climate.

### 3.3. Proximate Composition of Raw and Cooked Chicken Patties and Frozen Storage

The changes of chemical composition in raw and cooked chicken patties at zero time and after 3 months of storage at -20°C are given in Tables 3 and 4. At zero time, the moisture contents of raw and cooked chicken patties were decreased with higher addition ratio of CP and CS powder compared to control. At the end of storage period (3 months), the lowest moisture contents of raw chicken patties were observed with the samples containing 12% of CP or CS. These results are in agreement with Sharma and Yadav [56] who reported that the addition of pomegranate peel and aril bagasse powder caused a significant decrease in moisture content of chicken patties. Also, Mahdavi et al. [57] found that the moisture content of chicken burger was decreased in all chicken burger samples with increasing frozen storage time.

Also, the data in Tables 3 and 4 stated that the protein content was significantly higher in raw samples containing CS (3, 6, 9, and 12%) compared with the control sample at zero time, while higher protein content was observed with the sample containing 12% CP. Meanwhile, after 3 months of storage, the protein contents were significantly (*p* < 0.05) increased in all cooked samples compared with the same samples at zero time. These results are expected, since cantaloupe seeds powder had higher protein content (27.53%) than cantaloupe peel (7.50%) as indicated in Table 2. On opposite to our results, Sharma and Yadav [56] found that there were no significant (*p* > 0.05) differences in protein content of chicken meat incorporated with pomegranate peel and aril bagasse powder. Fat content in raw chicken patties was significantly increased with the increase of CS addition. The raw and cooked samples containing CS (12%) showed higher fat contents compared to raw and cooked control samples (Tables 3 and 4). Generally, frozen storage did not affect the fat content except in the sample containing 12% CS. This may be the result of cantaloupe seeds powder which are rich in lipids [52].

Furthermore, fiber content was significantly (*p* < 0.05) increased by increasing the addition ratio of CP and CS in raw and cooked samples at zero time and after frozen storage (3 months at -20°C). These results are in agreement with Mallek-Ayadi et al. [1] and da Cunha et al. [52], who reported that the cantaloupe peel and seeds flour were a good source of dietary fiber.

At zero time and after storage period (3 months), the ash content in raw and cooked chicken patties was significantly (*p* < 0.05) increased with the increasing ratio of CP and CS addition in all samples except the sample containing 3% CP compared with control (Tables 3 and 4). This could be due to the higher amount of ash of cantaloupe peel and seeds powder (Table 2). The ash contents of cooked control samples and some CS cooked samples (CS 3%, CS 6%, and CS 12%) were significantly increased during storage, while all CP samples (raw and cooked) were not changed during storage. These results are in agreement with Sharma and Yadav [56].

Concerning the carbohydrate contents in raw and cooked chicken patties samples (Tables 3 and 4), the results showed a significant (*p* < 0.05) decrease in carbohydrate (%) with the increasing ratio of CP and CS whether at zero time or after 3 months of storage at -20°C. On the whole, frozen storage had a significant effect on the carbohydrate content of both raw and cooked samples.

**Figure 1:**
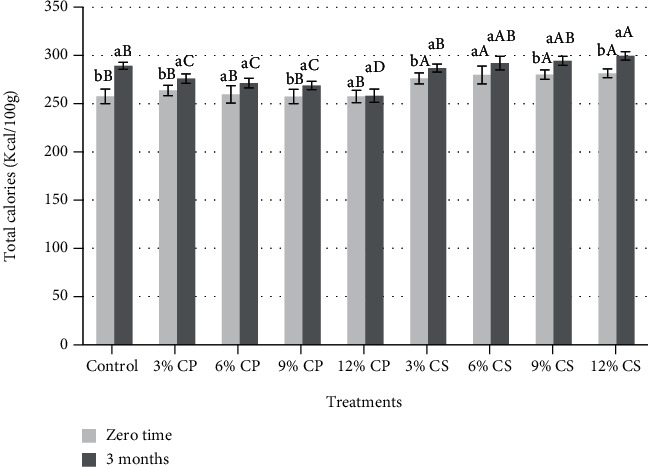
Changes in total calories of cooked chicken patties supplemented with cantaloupe peel (CP) and seeds (CS) powder during storage at -20 for 3 months.

Regarding to energy values in Figure 1, it was observed a significant (*p* < 0.05) increase in total calories (energy value) with the increasing in CP and CS addition in the cooked chicken patties either at zero time or after 3 months of frozen storage. Frozen storage had a significant (*p* < 0.05) effect on the energy values especially in cooked samples containing 3 and 9% CP and samples containing 3, 9, and 12% CS, whereas the energy values of all treatments were significantly (*p* < 0.05) increased by the end of the frozen storage period. These results were due to the decrease in moisture contents in frozen samples which resulted in an increase in protein and fat contents.

### 3.4. pH Value Determination

**Figure 2:**
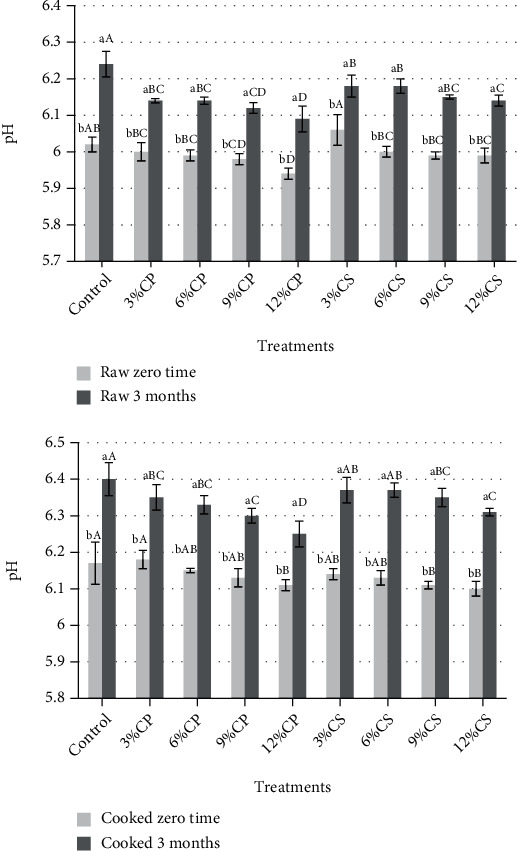
Changes in pH values of raw and cooked chicken patties supplemented with cantaloupe peel (CP) and seeds (CS) powder during storage at -20 for 3 months.

Results in Figure 2 indicated that at zero time, the pH values of raw patties (samples containing 9 and 12% CP and CS) and cooked patties (samples containing 12% CP and 9 and 12% CS) were significantly (*p* < 0.05) decreased compared to those of the control sample. Likewise, there was a significant decrease in all samples of raw patties and cooked patties samples containing 3, 6, 9, and 12% CP and 9 and 12% CS after 3 months of storage. Furthermore, the pH values had a significant (*p* < 0.05) increase after 3 months of frozen storage. The decrease in pH values with increasing ratio of the CP is due to the acidic nature of cantaloupe peel. Also, the increase in pH during frozen storage is due to the breakdown of protein, mainly amines. Similar findings were obtained by Chappalwar et al. [58]. They found that the addition of lemon peel powder to the chicken patties led to a decrease in the pH values, which may be attributed to the presence of polyphenols and flavonoids in lemon albedo as hesperidin, eriocitrin, and naringin.

### 3.5. Thiobarbituric Acid (TBA)

**Figure 3:**
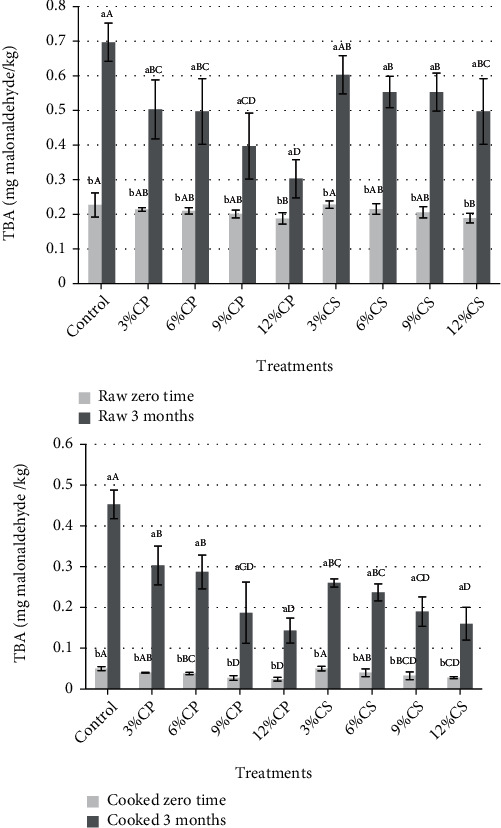
Changes in thiobarbituric acid (TBA) assay of raw and cooked chicken patties supplemented with cantaloupe peel (CP) and seeds (CS) powder during storage at -20 for 3 months.

The results in Figure 3 showed that at the zero time, the lower TBA values were found with raw patties samples containing 12% CP and CS and cooked samples containing 6, 9, and 12% CP and 9 and 12% CS compared to the control sample. Also, after 3 months of storage at -20°C, the raw and cooked patties samples containing CP and CS had lower TBA values compared to the control sample. This may be due to the effect of polyphenols and flavonoid compounds in CP and CS powder as antioxidant agents. Generally, TBA values in all patties samples were significantly (*p* < 0.05) increased with frozen storage (-20°C). This may be due to lipid oxidation and the formation of volatile basic nitrogen [59]. TBA values were within the permissible limits according to [60].

These findings are in agreement with Malav et al. [59], who found that the TBA values in all patties samples containing cabbage powder were significantly (*p* < 0.05) decreased compared to those in the control sample, which may be due to the presence of phenolic compounds in cabbage.

Also, Baioumy and Abedelmaksoud [61] found that formulating 5% orange peels (albedo) in the beef burgers has a positive impact; TBA values of the control and treatments were affected by the use of orange peel as there was a decrease in level of lipid oxidation, compared with the control sample. This confirms the positive effect of the orange peel on the quality characteristics and shelf life of beef burgers and reducing the microbial load during frozen storage.

### 3.6. Cooking Measurement of Chicken Patties

Cooking measurements, which include cooking loss, cooking yield, change of thickness, shrinkage, fat retention, and moisture retention, are one of the most important physical factors. Changes in quality during the burger meat cooking process are due to protein denaturation and the release of water and fat from the beef burger [62].

Table 5 shows the changes in water holding capacity (WHC), cooking loss, cooking yield, thickness, and shrinkage in different chicken patties treatments at zero time and after 3 months of storage at -20°C. WHC significantly (*p* < 0.05) increased by increasing the ratio of CP and CS addition as well as frozen storage in all chicken patties treatments. WHC was increased from 1.17 cm^2^/0.3 g in the control sample to 1.62 cm^2^ in the sample containing 12% CP at zero time and from 2.33 cm^2^/0.3 g to 2.79 cm^2^/0.3 g after frozen storage for 3 months for the same samples. This effect could be attributed to the presence of dietary fiber in CP. These results are in agreement with those reported by Serdaroğlu et al. [41] and Sharma and Yadav [56].

The increase in WHC values of chicken patties by frozen storage might be attributed to protein denaturation and loss of protein solubility [63].

Cooking loss was significantly (*p* < 0.05) decreased by increasing the ratio of cantaloupe peel and seeds powder addition; for example, the cooking loss was decreased from 18.07% with the control sample to 10.03% with the sample containing 12% CP at zero time and from 21.00% with the control sample to 12.19% with the sample containing 12% CP after frozen storage for 3 months. These results might be due to the ability of CP and CS to bind water and fat, which consequently decreased cooking loss. Haque et al. [64] found that the cooking loss of beef burger decreases with the addition of orange peel extract, and the cooking loss was increased at the beginning of storage and then decreased by the end of the storage period (after 60 days).

Data in Table 5 illustrated the cooking yield of chicken patties samples supplemented with CP and CS powder. The results indicated that the cooking yield was increased in all chicken patties samples containing CP and CS compared to the control sample at zero time and after frozen storage for 3 months. The decrement of cooking yield with frozen storage might be due to protein denaturation and loss of protein solubility which decreases water holding capacity consequently increasing moisture loss during cooking [63].

The change of diameter (shrinkage) was significantly (*p* < 0.05) decreased with an increasing ratio of cantaloupe peel and seeds powder. The higher shrinkage value was observed with the control samples at zero time and after frozen storage for 3 months (21.97% and 25.00%, respectively). Also, frozen storage had a significant effect on the shrinkage values which might be attributed to the ability of CP and CS to bind water and fat. Similar results were obtained by Bessar [65] who reported that increases in addition levels of orange and apple peels led to decreased shrinkage value in beef burgers.

No significant differences were observed in moisture retention among all treatments except samples containing 9 and 12% CP at zero time. Storage at -20°C for 3 months caused an increase in moisture retention for samples containing 3, 6, 9, and 12% of CP and 9 and 12% of CS. Also, findings indicated that fat retention was increased (*p* < 0.05) in sample patties containing 12% of CP and 3, 6, 9, and 12% of CS at zero time and after 3 months of storage at -20°C.

Meanwhile, reduction thickness was decreased (*p* < 0.05) by increasing the ratio of CP and CS in patty samples compared to the control samples at zero time and after 3 months of storage. Generally, frozen storage had an effect on moisture retention and shrinkage and thickness of patties, while it did not affect fat retention. These results were in line with Chappalwar et al. [58] who found that moisture and fat retention were increased with increasing levels of lemon albedo in chicken patties is due to the presence of fiber in lemon albedo, which has the ability to bind water and oil.

Hartmann et al. [66] who observed that the cooking yield and moisture retention were increased significantly in hamburger samples containing pumpkin peel flour (PPF). On the other hand, there was a decrease in the shrinkage percentage with the samples containing 3 and 4% PPF. This may be due to the presence of fiber in PPF which has the ability to interact with meat proteins by creating a network that prevents the transfer of water from the product to the surface, and this leads to an increase in cooking efficiency and reduces the shrinkage of the burger.

### 3.7. Antioxidant Activity of Chicken Patties

**Figure 4:**
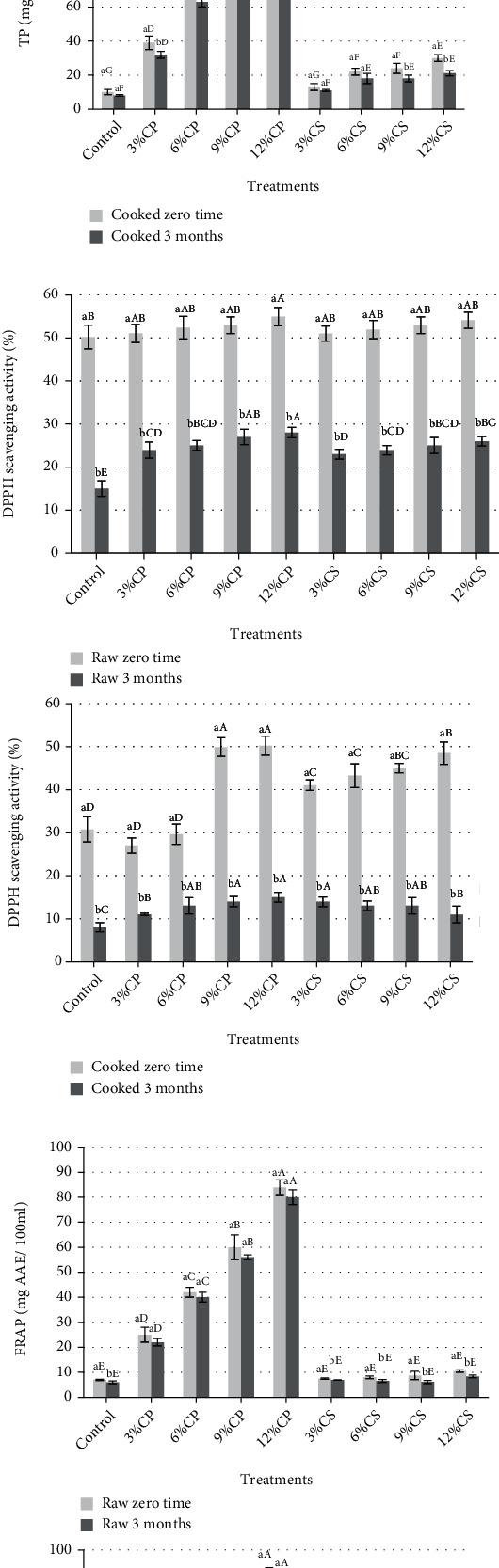
Changes in (a) total phenolic (TP), (b) DPPH scavenging activity, and (c) FRAP values of raw and cooked chicken patties supplemented with cantaloupe peel (CP) and seeds (CS) powder during storage at -20 for 3 months.

The antioxidant activity of raw and cooked patties during storage at -20°C for 3 months are shown in Figure 4. The addition of CP and CS to cooked patties formula increased the total phenolic contents (TP). The TP contents were significantly (*p* < 0.05) increased with the increase of CP and CS addition ratio. The addition of CP caused increased total phenolic contents compared the CS, and the best TP content was observed with the sample containing 12% CP. The findings revealed that at zero time, the DPPH radical scavenging activity (%) of raw and cooked patties significantly (*p* < 0.05) increased by increasing the ratio CP and CS powder addition and significantly (*p* < 0.05) decreased after 3 months of storage. Ferric reducing antioxidant power (FRAP) was significantly (*p* < 0.05) increased by increasing of CP addition ratio. The raw and cooked samples containing CP powder showed a higher FRAP value than the CS and control samples.

Total phenolic contents also showed a similar trend of antioxidant power (FRAP) that is increased significantly (*p* < 0.05) by increasing the CP ratio in patties samples for raw and cooked patties. It could be noted the total phenolic contents and FRAP were higher in cooked patties compared with raw patties; this may be due to the low moisture content after cooking and also the patties which retained the content of phenolic compounds after cooking which is an indicator that this product has health benefits for the consumer [30]. No significant differences were observed in FRAP values among all containing CS compared with the control sample at zero time, while the FRAP value decreased significantly (*p* < 0.05) after 3 months of storage in samples containing CS. Moreover, total phenolic contents decreased significantly (*p* < 0.05) with frozen storage especially in cooked sample patties containing 3, 6, and 9% CP and 9 and 12% CS.

### 3.8. Microbiological Quality

For microbial load, i.e., total bacterial count (TBC), coliform group bacteria, and yeast and mold count (log cfu/g) in raw and cooked patties treatments during storage at -20°C for 3 months, the findings indicated that at zero time, the TBC of patties samples was decreased with increasing the ratio of CP and CS addition compared with the control sample (Table 6), while, after 3 months of storage, the TBC was gradually increased. The increase in TBC after 3 months of storage may be due to an increase of amino acids and fatty acids resulting from the hydrolysis of proteins and fats during storage which is suitable for the growth of microorganisms. In general, the TBC after 3 months of storage is less than the permissible limit which is log10^7^ cfu/g for cooked meat products [67]. The coliforms were not detected in all patties samples at zero time and after 3 months of storage. This may be due to the high temperature during cooking that led to the destruction of the coliform bacteria. Similar results were obtained by Malav et al. [59]. Also, yeasts and molds were not detected in all patties treatments at zero time. Moreover, it could be observed that yeast and mold counts were decreased by increasing the ratio of CP and CS addition compared with the control sample after 3 months of storage. This may be due to the CP and CS which are rich sources of phenols and flavonoids, which have an antimicrobial effect [1].

### 3.9. Color of Raw and Cooked Chicken Patties Treatments as Affected by Addition Ratio of CP and CS Powder during Frozen Storage

Data presented in Tables 7 and 8 shows the color values (lightness, redness, and yellowness) of raw and cooked chicken patties treatments at zero time and after 3 months of frozen storage. It could be observed that there was a significant increase in lightness (*L*∗) with a steady increase in the percentage of CP or CS in raw and cooked chicken patties. The highest lightness value was observed in patties containing 12% CS in raw and cooked patties by the end of storage period. The increase in lightness could be attributed to the color of CP and CS. These results are in agreement with those reported by Chappalwar et al. [58] who observed a significant increase in lightness by increasing the ratio of lemon albedo powder in chicken patties.

Regarding redness (*a*∗) value, data revealed that using CP in raw and cooked chicken patties resulted in a significant increase in *a*∗ value compared to control sample during frozen storage period. On the other hand, the samples containing CS (3, 6, 9, and 12%) showed a significant (*p* < 0.05) decrease in redness after frozen storage for 3 months. However, there was a significant (*p* < 0.05) increase in redness value for cooked samples after frozen storage.

As for yellowness (*b*∗) value, the raw and cooked samples containing CP and CS were higher in yellowness value compared to the control sample, except raw and cooked patties samples containing 3% CS at zero time which showed the lowest yellowness value compared to control and other treatments. These findings might be because CP and CS were a good source of carotenoid pigments and polyphenol compounds. It can be noted that the frozen storage had a slight effect on the color parameters in the chicken patties.

Hartmann et al. [66] observed that hamburger containing 3% pumpkin peel flour had significantly increased lightness, redness, and yellowness which might be due to the presence of compounds in the peel like chlorophyll, carotenoids, and flavonoids, which are natural colorants in fruits and vegetables.

### 3.10. Texture Profile Analysis of Raw and Cooked Chicken Patties Treatments as Affected by Addition Ratio of CP and CS Powder during Frozen Storage

Table 9 shows the texture profile analysis (hardness, gumminess, chewiness, springiness cohesiveness, and resilience) of raw and cooked chicken patties during frozen storage for 3 months at -20°C. The hardness of raw chicken patties was significantly (*p* < 0.05) decreased by increasing the ratio of CP and CS addition, and the higher decrease was observed with samples containing 12% CP or 12% CS powder at zero times compared with the raw control sample. This may be due to the increase in dietary fiber in the CP and CS powder, as the increase in fiber gives a softer texture to the product. Also, the results showed a significant difference in hardness values among all treatments after 3 months of storage except samples containing 3% CP and 3 and 6% CS powder. Likewise, there was a significant (*p* < 0.05) decrease in hardness value among cooked samples containing 6, 9, and 12% CP and 9 and 12% CS after 3 months of storage. Generally, the hardness values were increased significantly (*p* < 0.05) with the increase in frozen storage time.

The gumminess of raw and cooked chicken patties samples significantly (*p* < 0.05) decreased with increasing the ratio of CP and CS powder addition through frozen storage (Table 9). In general, gumminess values were increased slightly with increasing the frozen storage time. Similar results were obtained by Chappalwar et al. [58] who observed a decrease in hardness, gumminess, cohesiveness, and springiness values of chicken patties by increasing the ratio of lemon albedo powder. They attributed this to the effect of lemon albedo on the protein system, the presence of water, and the binding of fats in the lemon peel which provides a smooth texture.

Chewiness value also showed a similar trend of resilience that is increased with increasing the CP and CS ratio in patties samples. Moreover, during frozen storage, chewiness values were increased significantly (*p* < 0.05) expect samples containing 6 and 12% of CP powder. However, there was no significant (*p* > 0.05) difference in resilience value of raw and cooked samples after 3 months of frozen storage. The storage times did not affect the resilience value of all treatments. No significant (*p* > 0.05) differences were observed in springiness value among the control and samples containing CP and CS powder during frozen storage. On the other hand, the springiness values of cooked samples containing CP powder were significantly (*p* < 0.05) decreased during frozen storage, while the higher springiness values by the end of storage were found with samples containing CS powder. Cohesiveness values of both raw and cooked patties were significantly (*p* < 0.05) lower with samples containing 3, 6, 9, and 12% of CP and 12% of CS at zero time. Moreover, there was a significant (*p* < 0.05) decrease in samples containing 9 and 12% CP and 12% CS of raw patties and 12% CS in cooked patties after 3 months of storage.

Cohesiveness values among samples containing 9 and 12% CP were significantly (*p* < 0.05) increased after 3 months of frozen storage. Sharma and Yadav [56] found that incorporation of pomegranate peel powder (PPP) in chicken patties led to significant increase in hardness and gumminess, while chewiness value was lower in PPP-treated patties.

### 3.11. Sensory Evaluation

**Figure 5:**
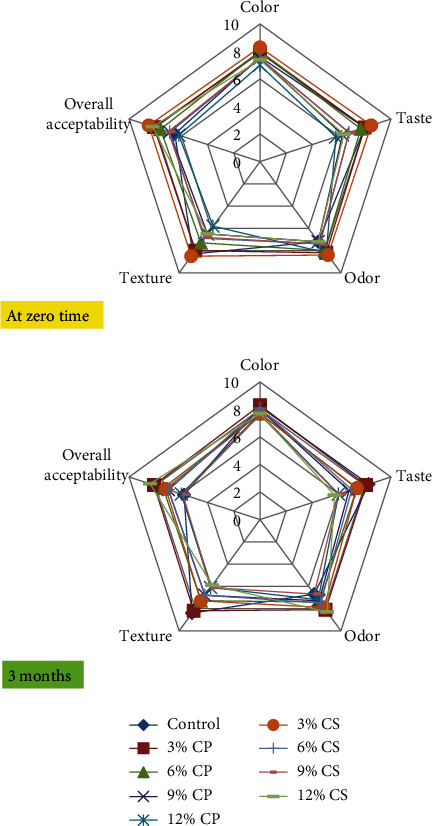
Changes in sensory evaluation of chicken patties supplemented with cantaloupe peel (CP) and seeds (CS) powder during storage at -20 for 3 months.

Sensorial evaluation of chicken patties is depicted in Figure 5. There were no significant (*p* > 0.05) differences in the color score found between the control sample and chicken patties containing CP and CS powder. Taste score was significantly (*p* < 0.05) decreased in patties samples containing 9 and 12% CP and 6, 9, and 12% of CS. This may be due to the association of the taste with high phenolic compounds in the peel, which caused a slightly bitter and acidic taste to the patties [58]. Odor and overall acceptability scores of 3% CS were significantly (*p* < 0.05) higher than those of the control sample. Moreover, there was a significant difference (*p* < 0.05) in texture score between control and samples containing 6, 9, and 12% CP and CS powder. The frozen storage had a negative effect on most sensory characteristics of among treatments. It was observed that the sample containing 3% CS was significantly (*p* < 0.05) decreased in texture and overall acceptability scores after 3 months of frozen storage. Hartmann et al. [66] observed that there were no significant (*p* > 0.05) differences in appearance, texture, color, and purchase intention between the control sample and hamburger containing at 1, 2, 3, and 4% pumpkin peel flour (PPF), while the control sample and sample containing 1% PPF were more accepted than 4% PPF in regard to overall acceptance. This may be due to the presence of phenols in the peel of vegetables, like tannins, and this enhances the astringent taste.

## 4. Conclusion

This study was carried out to improve the quality, cooking properties, and antioxidant activity of chicken patties by the addition of cantaloupe (peel and seeds) powder which was considered as a good source of phytochemical components, crude fibers, protein, fat, and minerals. The use of cantaloupe (peel and seeds) powder improved the functionality, quality properties, and antioxidant activity of chicken patties. Also, the results showed that the addition of CP and CS caused a significant (*p* < 0.05) increase in cooking yield, fat retention, and moisture retention. In addition, chicken patty samples had a high microbiological quality compared to the control sample. Chicken patties fortified with CP and CS powder at a ratio of 3% showed the best overall acceptability compared with the control sample and other treatments. Generally, this study recommended the use of CP and CS at a ratio of 9% in the development of meat products' with good functional properties and acceptability.

## Figures and Tables

**Table 1:** Formula of chicken patties (g) prepared with different ratios of cantaloupe peel and seeds powder.

Ingredients (%)	Treatments								
	Control	3% CP	6% CP	9% CP	12% CP	3% CS	6% CS	9% CS	12% CS
Chicken meat	80.00	80.00	80.00	80.00	80.00	80.00	80.00	80.00	80.00
Whole eggs	6.80	6.80	6.80	6.80	6.80	6.80	6.80	6.80	6.80
Bread crumbs	12.00	9.00	6.00	3.00	—	9.00	6.00	3.00	—
Cantaloupe peels powder (CP)	—	3.00	6.00	9.00	12.00	—	—	—	—
Cantaloupe seed powder (CS)	—	—	—	—	—	3.00	6.00	9.00	12.00
Spices mixture	0.2	0.2	0.2	0.2	0.2	0.2	0.2	0.2	0.2
Salt	1.00	1.00	1.00	1.00	1.00	1.00	1.00	1.00	1.00

CP: cantaloupe peel powder; CS: cantaloupe seed powder. Spice mixture: ground black pepper, ground cumin, and onion powder.

**Table 2:** Physiochemical analysis, mineral content, functional properties, and antioxidant activity of cantaloupe peel (CP) and seed (CS) powder.

Component	CP powder	CS powder
Moisture (%)	17.99±0.27^a^	7.20±0.11^b^
Fat (%)	2.13±0.13^b^	29.54±0.71^a^
Protein (%)	7.50±0.51^b^	27.53±1.09^a^
Total carbohydrate (%)	68.8±3.83^a^	31.10±1.92^b^
Total dietary Fiber (%)	39.33±2.08^a^	24.17±1.61^b^
Ash (%)	3.54±0.13^b^	4.64±0.14^a^
pH	6.22±0.02^b^	7.00±0.11^a^

Minerals (mg/ 100g)		
Calcium	1000±1.53^a^	940±2.52^b^
Potassium	760±1.53^a^	410±1.37^b^
Magnesium	320±1.05^b^	900±1.53^a^
Iron	1.60±0.10^b^	2.50±0.09^a^
Zinc	0.35±0.02^b^	2.10±0.10^a^
Sodium	130±1.95^b^	289±1.05^a^

Functional properties		
Water retention capacity (g water/g)	5.96±0.12^a^	4.43±0.17^b^
Oil retention capacity (g oil/g)	2.35±0.05^a^	2.50±0.10^a^

Antioxidant Activity		
Total phenolic (mg GAE/100 mL)	1050±50.95^a^	220±19.90^b^
DPPH scavenging (%)	91.34±3.89^a^	49.04±3.96^b^
FRAP (mg AAE/ 100 mL)	700±55.50^a^	25.03±2.05^b^

All determinations were carried out in triplicate and mean value ± SD. Different letters in rows indicate significant different value at *p* < 0.05.

**Table 3:** Changes in proximate chemical composition of raw chicken patties supplemented with cantaloupe peel (CP) and seed (CS) powder during storage at -20 for 3 months.

Treatments	Storage time (month)	Moisture (%)	Protein (%)	Fat (%)	Ash (%)	Total carbohydrates (%)	Dietary fiber (%)
Control	0	62.97±1.93^aA^	20.16±1.11^bC^	10.17±0.76^aD^	1.70±0.08^aF^	2.67±0.06^aB^	2.34±0.35^bF^
	3	58.45±1.87^bAB^	23.67±1.15^aABC^	11.00±0.89^aE^	1.78±0.03^aF^	2.08±0.01^bC^	3.01±0.23^aF^

3% CP	0	61.79±2.28^aA^	20.31±1.59^aBC^	10.23±0.75^aD^	1.80±0.03^aF^	2.85±0.04^aA^	3.03±0.25^bE^
	3	58.64±2.51^aA^	21.76±1.02^aCD^	11.23±0.64^aDE^	1.90±0.10^aEF^	2.47±0.03^bB^	4.00±0.29^aE^

6% CP	0	59.97±2.93^aAB^	21.26±1.33^aABC^	10.33±0.67^aD^	1.90±0.06^aE^	2.48±0.08^bC^	4.05±0.20^bD^
	3	56.50±1.65^aAB^	22.42±1.14^aBCD^	11.35±0.65^aDE^	2.00±0.05^aDE^	2.63±0.03^aA^	5.10±0.25^aD^

9% CP	0	57.64±2.51^aBC^	21.63±1.19^aABC^	10.55±0.85^aD^	2.03±0.09^aD^	2.14±0.13^aD^	6.01±0.31^bB^
	3	54.44±3.50^aAB^	23.72±1.16^aABC^	11.60±0.80^aDE^	2.13±0.16^aCD^	1.09±0.01^bD^	7.01±0.21^aB^

12% CP	0	56.03±2.28^aBC^	21.26±1.57^aABC^	10.89±0.89^aCD^	2.21±0.07^aC^	1.13±0.08^aG^	8.49±0.23^bA^
	3	52.74±3.26^aB^	21.12±1.47^aD^	11.92±0.23^aCDE^	2.31±0.18^aBC^	0.91±0.07^bE^	11.01±0.32^aA^

3% CS	0	59.11±2.79^aAB^	22.80±1.29^aAB^	11.42±0.73^aCD^	1.99±0.01^aD^	1.98±0.03^aE^	2.70±0.20^bEF^
	3	56.11±1.13^aAB^	24.16±1.01^aAB^	12.53±0.55^aCD^	2.13±0.16^aCD^	1.06±0.02^bD^	4.00±0.28^aE^

6% CS	0	57.07±1.31^aBC^	23.43±1.25^aA^	12.17±0.93^aBC^	2.21±0.03^bC^	1.91±0.09^aE^	3.21±0.29^bE^
	3	54.13±1.73^aAB^	24.98±1.09^aA^	13.17±0.95^aBC^	2.35±0.05^aB^	1.07±0.01^bD^	4.31±0.29^aE^

9% CS	0	55.97±1.48^aBC^	23.49±1.26^aA^	12.88±0.88^aAB^	2.38±0.03^aB^	1.34±0.05^aF^	3.93±0.46^bD^
	3	53.44±3.05^aAB^	24.15±1.01^aAB^	13.90±0.91^aAB^	2.40±0.03^aAB^	1.09±0.02^bD^	5.01±0.31^aD^

12% CS	0	54.86±1.06^aC^	22.92±1.21^aA^	13.65±0.65^aA^	2.49±0.02^aA^	1.28±0.07^aF^	4.80±0.40^bC^
	3	52.78±5.64^aB^	22.87±1.28^aABCD^	14.68±0.68^aA^	2.59±0.09^aA^	1.07±0.02^bD^	6.01±0.32^aC^

Mean values (±SD); means followed by different capital letters in the same column (effect of treatments) are significant by Duncan's multiple test (*p* < 0.05). Means followed by different small letters in the same column (effect of storage time) are significant by Duncan's multiple test (*p* < 0.05).

**Table 4:** Changes in proximate chemical composition of cooked chicken patties supplemented with cantaloupe peel (CP) and seed (CS) powder during storage at -20 for 3 months.

Treatments	Storage time (month)	Moisture (%)	Protein (%)	Fat (%)	Ash (%)	Total carbohydrates (%)	Dietary fiber (%)
Control	0	43.20±9.72^aA^	24.11±1.11^bF^	8.67±0.58^aD^	1.74±0.13^bE^	20.78±2.03^aA^	1.497±0.20^aG^
	3	35.19±1.48^aA^	27.78±1.10^aE^	9.77±0.75^aDE^	2.81±0.05^aA^	22.52±2.03^aA^	1.93±0.45^aF^

3% CP	0	39.12±2.53^aAB^	26.76±0.96^bE^	8.70±0.63^aD^	1.82±0.19^aDE^	19.60±1.80^aAB^	4.00±0.27^bEF^
	3	35.03±1.96^aA^	30.34±1.76^aDE^	8.91±0.91^aE^	1.97±0.13^aE^	18.63±1.47^aB^	5.10±0.25^aE^

6% CP	0	38.97±2.03^aAB^	28.06±1.48^bDE^	8.84±0.76^aCD^	1.96±0.13^aCDE^	16.96±1.45^aC^	5.207±0.29^bD^
	3	35.10±1.77^aA^	32.80±1.49^aDC^	9.05±0.96^aDE^	2.08±0.01^aE^	14.66±1.85^aC^	6.31±0.12^aD^

9% CP	0	37.77±2.25^aAB^	30.82±1.83^bBC^	9.07±0.90^aCD^	2.10±0.11^aBC^	13.14±1.02^aDE^	7.11±0.22^bB^
	3	33.81±2.60^aA^	34.07±1.27^aBC^	9.78±0.93^aDE^	2.19±0.06^aD^	11.15±1.35^aEF^	9.01±0.21^aB^

12% CP	0	35.03±1.98^aB^	31.81±0.99^bAB^	9.37±0.55^aCD^	2.3±0.20^aAB^	11.79±1.07^aE^	10.01±0.32^bA^
	3	31.21±1.95^aA^	34.99±1.12^aBC^	9.93±0.55^aDE^	2.38±0.03^aC^	7.19±0.97^bG^	14.29±0.39^aA^

3% CS	0	37.77±2.25^aAB^	29.16±0.77^bCD^	9.93±0.70^aBC^	2.03±0.09^bCD^	17.56±1.50^aBC^	3.53±0.45^bF^
	3	34.24±2.24^aA^	34.43±1.83^aBC^	10.53±0.55^aCD^	2.22±0.03^aD^	13.57±1.33^bCD^	5.00±0.28^aE^

6% CS	0	36.98±2.23^aAB^	30.55±1.64^bBC^	10.64±0.41^aAB^	2.27±0.06^bAB^	15.47±1.58^aCD^	4.10±0.25^bE^
	3	33.30±1.31^bA^	35.10±1.65^aBC^	11.43±0.45^aBC^	2.44±0.05^aC^	12.20±1.04^bDE^	5.53±0.45^aE^

9% CS	0	36.64±2.36^aAB^	32.19±1.05^bAB^	11.10±0.17^aA^	2.42±0.03^aA^	12.86±1.03^aE^	4.80±0.40^bD^
	3	33.15±2.50^aA^	36.83±1.67^aAB^	12.33±0.85^aAB^	2.49±0.08^aC^	9.00±0.80^bFG^	6.20±0.30^aD^

12% CS	0	35.03±1.98^aB^	33.72±1.41^bA^	11.28±0.54^bA^	2.49±0.08^bA^	11.28±1.08^aE^	6.21±0.29^bC^
	3	31.75±2.88^aA^	38.20±1.43^aA^	13.04±0.96^aA^	2.67±0.07^aB^	7.34±0.65^bG^	7.01±0.22^aC^

Mean values (±SD); means followed by different capital letters in the same column (effect of treatments) are significant by Duncan's multiple test (*p* < 0.05). Means followed by different small letters in the same column (effect of storage time) are significant by Duncan's multiple test (*p* < 0.05).

**Table 5:** Changes in water holding capacity (%) and cooking properties of cooked chicken patties supplemented with cantaloupe peel (CP) and seed (CS) powder during storage at -20 for 3 months.

Treatments	Storage time (month)	Water holding capacity (cm^2^/0.3 g)	Cooking loss (%)	Cooking yield (%)	Change of thickness (%)	Change of shrinkage (%)	Fat retention (%)	Moisture retention (%)
Control	0	1.17±0.08^bC^	18.07±0.70^aA^	81.94±2.42^aD^	48.00±2.05^bA^	21.97±1.95^aA^	69.00±3.95^aD^	66.00±5.95^aC^
	3	2.33±0.14^aC^	21.00±1.91^aA^	79.00±1.91^aD^	58.90±1.40^aA^	25.00±1.91^aA^	70.00±2.05^aE^	39.00±3.95^bF^

3% CP	0	1.29±0.09^bBC^	15.07±1.25^bBCD^	84.93±1.25^aBCD^	45.30±3.51^bA^	15.00±1.90^aBC^	70.00±3.05^aCD^	70.00±4.95^aABC^
	3	2.37±0.18^aBC^	17.77±1.25^aABC^	82.23±2.25^aBCD^	52.03±2.05^aBC^	16.97±0.95^aC^	71.00±1.95^aE^	45.00±2.95^bCDE^

6% CP	0	1.30±0.10^bBC^	14.07±1.31^aCD^	85.93±1.92^aBC^	36.30±2.52^bC^	11.97±1.95^aDE^	71.03±2.05^aCD^	74.00±3.05^aABC^
	3	2.48±0.13^aBC^	16.33±1.25^aBCD^	83.67±2.25^aBC^	45.07±3.10^aD^	13.97±1.05^aD^	73.00±2.95^aDE^	49.00±3.05^bBC^

9% CP	0	1.47±0.12^bAB^	12.03±0.81^aE^	87.97±1.72^aAB^	30.30±1.53^bD^	8.03±0.55^bF^	73.07±2.10^aCD^	76.00±3.95^aAB^
	3	2.60±0.10^aAB^	14.22±2.23^aDE^	85.78±2.23^aAB^	35.97±2.95^aE^	10.03±0.95^aF^	75.00±2.95^aCDE^	52.00±1.95^bAB^

12% CP	0	1.62±0.12^bA^	10.03±0.55^aF^	89.97±1.38^aA^	21.03±1.05^bE^	5.40±0.41^bG^	75.00±2.95^aBC^	78.00±4.95^aA^
	3	2.79±0.16^aA^	12.19±2.20^aE^	87.81±2.20^aA^	24.97±1.05^aF^	7.03±0.55^aG^	76.90±1.90^aBCD^	55.00±2.95^bA^

3% CS	0	1.18±0.09^bC^	16.77±0.87^aAB^	83.23±2.40^aCD^	45.07±2.90^bA^	16.97±0.95^bB^	75.00±3.95^aBC^	68.00±4.85^aBC^
	3	2.32±0.13^aC^	19.23±2.04^aAB^	80.77±2.04^aCD^	54.97±3.95^aAB^	20.23±0.68^aB^	79.00±3.95^aBC^	41.00±3.95^bEF^

6% CS	0	1.25±0.16^bBC^	15.73±1.62^aBC^	84.27±2.19^aCD^	40.97±2.45^bB^	15.97±0.95^bB^	79.00±1.96^aAB^	69.00±3.95^aBC^
	3	2.33±0.12^aC^	18.03±1.96^aABC^	81.97±1.96^aBCD^	49.97±2.95^aC^	17.97±0.95^aC^	81.00±2.95^aAB^	43.00±2.95^bDEF^

9% CS	0	1.32±0.11^bBC^	14.03±0.95^aCD^	85.97±1.70^aBC^	36.03±1.05^bC^	12.97±1.00^aCD^	82.00±2.95^aA^	70.00±3.05^aABC^
	3	2.40±0.11^aBC^	16.10±1.50^aBCD^	83.90±2.18^aBC^	44.97±1.95^aD^	14.97±0.95^aD^	85.00±4.90^aA^	45.00±2.65^bCDE^

12% CS	0	1.45±0.16^bAB^	13.27±0.64^aED^	86.73±1.55^aABC^	28.93±1.40^bD^	10.30±0.61^aE^	84.00±3.95^aA^	72.00±2.05^aABC^
	3	2.55±0.11^aBC^	15.33±1.35^aCDE^	85.00±1.48^aAB^	40.97±2.55^aD^	11.97±0.95^aE^	86.00±2.95^aA^	47.00±2.95^bBCD^

Mean values (±SD); means followed by different capital letters in the same column (effect of treatments) are significant by Duncan's multiple test (*p* < 0.05). Means followed by different small letters in the same column (effect of storage time) are significant by Duncan's multiple test (*p* < 0.05).

**Table 6:** Changes in microbiological quality of raw and cooked chicken patties supplemented with cantaloupe peel (CP) and seeds (CS) powder during storage at -20 for 3 months.

Treatments	Chicken patties	Storage time (months)	Total bacterial count (log cfu/g)	Yeast and mold count (log cfu/g)	Total coliform (log cfu/g)
Control	Raw	0	2.16 ± 0.14^bA^	ND	ND
		3	5.00 ± 0.11^aA^	3.00 ± 0.11^A^	ND
	Cooked	0	0.537 ± 0.015^bA^	ND	ND
		3	1.25 ± 0.05^aA^	1.51 ± 0.110^A^	ND
3% CP	Raw	0	1.81 ± 0.02^bB^	ND	ND
		3	4.43 ± 0.06^aB^	2.52 ± 0.13^B^	ND
	Cooked	0	0.440 ± 0.017^bB^	ND	ND
		3	1.01 ± 0.19^aB^	0.807 ± 0.021^B^	ND
6% CP	Raw	0	1.81 ± 0.08^bB^	ND	ND
		3	4.00 ± 0.11^aC^	2.23 ± 0.06^C^	ND
	Cooked	0	0.433 ± 0.012^bB^	ND	ND
		3	1.00 ± 0.02^aB^	0.403 ± 0.055^D^	ND
9% CP	Raw	0	1.70 ± 0.01^bBC^	ND	ND
		3	3.00 ± 0.11^aD^	2.23 ± 0.25^C^	ND
	Cooked	0	0.427 ± 0.015^bB^	ND	ND
		3	0.807 ± 0.020^aC^	0.237 ± 0.032^E^	ND
12% CP	Raw	0	1.60 ± 0.11^bC^	ND	ND
		3	2.52 ± 0.08^aE^	0.000 ± 0.000^E^	ND
	Cooked	0	0.403 ± 0.055^bBC^	ND	ND
		3	0.757 ± 0.042^aCD^	0.000 ± 0.000^F^	ND
3% CS	Raw	0	1.70 ± 0.11^aBC^	ND	ND
		3	1.60 ± 0.11^aF^	2.52 ± 0.11^B^	ND
	Cooked	0	0.407 ± 0.055^bBC^	ND	ND
		3	0.737 ± 0.015^aCD^	0.603 ± 0.095^C^	ND
6% CS	Raw	0	1.60 ± 0.01^aC^	ND	ND
		3	1.00 ± 0.09^bG^	2.11 ± 0.12^C^	ND
	Cooked	0	0.390 ± 0.060^bBCD^	ND	ND
		3	0.603 ± 0.095^aDE^	0.283 ± 0.047^E^	ND
9% CS	Raw	0	1.40 ± 0.06^aD^	ND	ND
		3	1.00 ± 0.09^bG^	1.70 ± 0.11^D^	ND
	Cooked	0	0.350 ± 0.030^bCD^	ND	ND
		3	0.563 ± 0.095^aDE^	0.233 ± 0.031^E^	ND
12% CS	Raw	0	1.40 ± 0.05^aD^	ND	ND
		3	1.00 ± 0.10^bG^	1.60 ± 0.11^D^	ND
	Cooked	0	0.330 ± 0.030^bD^	ND	ND
		3	0.503 ± 0.095^aE^	0.000 ± 0.000^F^	ND

Mean values (±SD); means followed by different capital letters in the same column (effect of treatments) are significant by Duncan's multiple test (*p* ≤ 0.05). Means followed by different small letters in the same column (effect of storage time) are significant by Duncan's multiple test (*p* ≤ 0.05).

**Table 7:** Changes in color values of raw chicken patties supplemented with cantaloupe peel (CP) and seeds (CS) powder during storage at -20 for 3 months.

Treatments	Storage time (month)	Lightness (*L*∗)	Redness (*a*∗)	Yellowness (*b*∗)
Control	0	39.52 ± 1.48^bF^	6.03 ± 0.27^aD^	19.83 ± 1.23^aCD^
	3	41.73 ± 1.45^aE^	5.77 ± 0.62^aC^	15.20 ± 1.13^bC^
3%CP	0	41.60 ± 1.91^aEF^	7.21 ± 0.41^aC^	23.76 ± 2.25^aAB^
	3	42.85 ± 1.65^aE^	6.10 ± 0.89^bBC^	21.78 ± 1.85^aAB^
6%CP	0	42.86 ± 1.15^aE^	7.88 ± 0.39^aB^	24.00 ± 2.00^aA^
	3	43.73 ± 1.50^aE^	6.10 ± 0.87^bBC^	22.25 ± 1.18^aAB^
9%CP	0	45.85 ± 1.25^aD^	8.12 ± 0.32^aB^	24.14 ± 1.85^aA^
	3	47.53 ± 1.15^aD^	6.56 ± 0.39^bAB^	23.32 ± 1.91^aA^
12%CP	0	49.58 ± 1.55^aC^	8.66 ± 0.26^aA^	24.32 ± 1.52^aA^
	3	51.86 ± 1.31^aC^	6.85 ± 0.79^bA^	23.50 ± 1.08^aA^
3%CS	0	53.74 ± 1.73^aB^	5.59 ± 0.20^aDE^	19.34 ± 1.45^aD^
	3	55.93 ± 1.05^aB^	4.58 ± 0.34^bD^	17.49 ± 1.93^aC^
6%CS	0	55.22 ± 1.69^aAB^	5.57 ± 0.14^aDE^	20.64 ± 1.66^aBCD^
	3	57.24 ± 1.04^aAB^	4.63 ± 0.35^bD^	20.21 ± 1.10^aB^
9%CS	0	56.46 ± 1.03^aA^	5.54 ± 0.15^aDE^	21.80 ± 1.20^aABCD^
	3	57.93 ± 1.10^aAB^	4.69 ± 0.32^bD^	20.86 ± 1.80^aAB^
12%CS	0	56.97 ± 1.76^aA^	5.52 ± 0.17^aE^	22.76 ± 1.97^aABC^
	3	59.30 ± 1.70^aA^	4.85 ± 0.27^bD^	20.91 ± 1.34^aAB^

Mean values (±SD); means followed by different capital letters in the same column (effect of treatments) are significant by Duncan's multiple test (*p* ≤ 0.05). Means followed by different small letters in the same column (effect of storage time) are significant by Duncan's multiple test (*p* ≤ 0.05).

**Table 8:** Changes in color values of cooked chicken patties supplemented with cantaloupe peel (CP) and seeds (CS) powder during storage at -20 for 3 months.

Treatments	Storage time (month)	Lightness (*L*∗)	Redness (*a*∗)	Yellowness (*b*∗)
Control	0	43.36 ± 1.27^aE^	7.98 ± 0.43^aC^	21.46 ± 1.60^aC^
	3	44.79 ± 1.57^aG^	6.63 ± 0.44^bE^	20.91 ± 1.77^aB^
3%CP	0	46.19 ± 1.82^aD^	8.90 ± 0.42^aB^	24.86 ± 1.05^aB^
	3	47.64 ± 1.85^aFG^	8.40 ± 0.40^aAB^	23.86 ± 1.08^aA^
6%CP	0	47.64 ± 1.37^aCD^	9.49 ± 0.41^aAB^	26.30 ± 1.50^aAB^
	3	48.10 ± 2.00^aEF^	8.27 ± 0.44^aABC^	24.04 ± 1.28^aA^
9%CP	0	50.22 ± 1.23^aBC^	9.11 ± 0.51*a*^B^	27.24 ± 1.55^aAB^
	3	51.17 ± 1.80^aE^	8.69 ± 0.30^aAB^	24.66 ± 1.93^aA^
12%CP	0	52.58 ± 1.68^bB^	10.19 ± 0.51^aA^	28.50 ± 1.50^aA^
	3	57.34 ± 2.25^aD^	9.21 ± 0.68^aA^	24.91 ± 1.81^aA^
3%CS	0	57.99 ± 1.03^aA^	6.95 ± 0.25^aD^	20.87 ± 1.05^bC^
	3	59.79 ± 1.80^aCD^	7.13 ± 0.50^aDE^	24.19 ± 1.10^aA^
6%CS	0	58.55 ± 1.78^aA^	7.14 ± 0.45^aD^	22.13 ± 1.85^aC^
	3	61.89 ± 2.11^aBC^	7.24 ± 0.35^aCDE^	24.39 ± 1.70^aA^
9%CS	0	59.55 ± 1.64^bA^	7.55 ± 0.26^aCD^	25.23 ± 1.75^aB^
	3	64.64 ± 1.25^aAB^	7.91 ± 0.25^aBCD^	24.86 ± 1.93^aA^
12%CS	0	60.62 ± 1.23^bA^	7.99 ± 0.49^aC^	28.91 ± 1.11^aA^
	3	67.34 ± 2.25^aA^	7.96 ± 0.62^aBCD^	25.14 ± 1.06^bA^

Mean values (±SD); means followed by different capital letters in the same column (effect of treatments) are significant by Duncan's multiple test (*p* ≤ 0.05). Means followed by different small letters in the same column (effect of storage time) are significant by Duncan's multiple test (*p* ≤ 0.05).

**Table 9:** Changes in texture profile analysis of raw and cooked chicken patties supplemented with cantaloupe peel (CP) and seed (CS) powder during storage at -20 for 3 months.

Treatments	Chicken patties	Storage (months)	Hardness (g/s)	Springiness (mm)	Cohesiveness (ratio)	Gumminess (g/s)	Chewiness (mJ)	Resilience
Control	raw	0	334±24.6^bA^	0.830±0.027^aA^	0.440±0.040^aA^	293±1.2^aA^	6.33±0.15^bG^	0.110±0.010^aC^
		3	1300±100.0^aA^	0.827±0.025^aA^	0.467±0.015^aA^	327±2.5^aA^	7.33±0.15^aG^	0.123±0.006^aD^
	cooked	0	431±30.6^bA^	0.667±0.015^aC^	0.377±0.025^aA^	400±1.0^aA^	7.17±0.29^bE^	0.160±0.010^aD^
		3	1400±100.0^aA^	0.647±0.006^aD^	0.390±0.010^aA^	420±2.7^aA^	8.23±0.25^aG^	0.170±0.020^aD^

3% CP	raw	0	330±27.8^bAB^	0.830±0.027^aA^	0.420±0.020^aA^	233±1.5^aB^	6.53±0.15^bG^	0.183±0.015^aA^
		3	1210±36.1^aABC^	0.817±0.015^aA^	0.443±0.040^aAB^	270±2.7^aB^	7.60±0.17^aG^	0.190±0.010^aAB^
	cooked	0	428±25.5^bA^	0.757±0.021^aAB^	0.357±0.021^aA^	317±2.1^bB^	8.23±0.25^bD^	0.207±0.012^aC^
		3	1283±76.4^aAB^	0.667±0.015^bCD^	0.360±0.027^aAB^	373±2.1^aB^	10.30±0.27^aEF^	0.213±0.023^aC^

6% CP	raw	0	310±26.5^bABC^	0.817±0.015^aA^	0.420±0.020^aA^	220±1.0^bBC^	7.07±0.12^bF^	0.183±0.015^aA^
		3	1177±25.2^aBC^	0.797±0.015^aA^	0.443±0.040^aAB^	263±1.5^aBC^	8.10±0.10^aF^	0.190±0.010^aAB^
	cooked	0	422±23.1^bA^	0.753±0.015^aAB^	0.363±0.015^aA^	243±2.1^aC^	10.30±0.27^aC^	0.220±0.020^aBC^
		3	1250±50.0^aB^	0.673±0.021^bCD^	0.377±0.025^aA^	263±1.5^aC^	10.60±0.31^aE^	0.230±0.027^aBC^

9% CP	raw	0	287±20.2^bBC^	0.837±0.015^aA^	0.393±0.012^aABC^	207±1.2^bCD^	7.57±0.21^bE^	0.150±0.020^aB^
		3	1143±40.4^aCD^	0.817±0.015^aA^	0.403±0.032^aBCD^	237±1.2^aBCD^	8.63±0.15^aE^	0.160±0.010^aC^
	cooked	0	417±22.0^bA^	0.763±0.015^aAB^	0.317±0.015^bB^	217±1.5^aCD^	12.30±0.31^bB^	0.220±0.020^aBC^
		3	1233±57.7^aB^	0.690±0.010^bBC^	0.357±0.021^aAB^	230±1.0^aD^	13.00±0.32^aC^	0.230±0.027^aB^C

12% CP	raw	0	285±22.9^bBC^	0.830±0.020^aA^	0.360±0.017^aBC^	167±1.5^bEF^	8.23±0.25^bD^	0.143±0.025^aB^
		3	943±81.5^aE^	0.820±0.020^aA^	0.383±0.021^aCD^	193±0.6^aEF^	9.23±0.25^aD^	0.153±0.015^aC^
	cooked	0	410±21.8^bA^	0.763±0.015^aAB^	0.273±0.015^bC^	217±1.5^aCD^	14.80±0.27^aA^	0.270±0.020^aA^
		3	1043±92.9^aC^	0.690±0.010^bBC^	0.333±0.015^aB^	230±1.0^aD^	15.00±0.15^aA^	0.287±0.015^aA^

3% CS	raw	0	324±22.7^bAB^	0.830±0.020^aA^	0.410±0.036^aAB^	203±0.6^bCD^	7.43±0.40^bEF^	0.150±0.020^aB^
		3	1280±26.5^aAB^	0.817±0.015^aA^	0.433±0.029^aABC^	267±1.5^aBC^	8.40±0.36^aEF^	0.160±0.010^aC^
	cooked	0	424±23.3^bA^	0.727±0.025^aB^	0.367±0.015^aA^	187±2.3^aDE^	8.47±0.45^bD^	0.190±0.010^aCD^
		3	1340±52.9^aAB^	0.713±0.032^aAB^	0.383±0.015^aA^	217±1.5^aDE^	10.00±0.50^aF^	0.207±0.012^aCD^

6% CS	raw	0	317±23.7^bAB^	0.840±0.010^aA^	0.410±0.036^aAB^	200±2.0^aCD^	9.17±0.29^bC^	0.160±0.010^aAB^
		3	1204±21.4^aABC^	0.817±0.015^aA^	0.433±0.029^aABC^	230±2.7^aCDE^	10.30±0.21^aC^	0.170±0.010^aBC^
	cooked	0	421±23.5^bA^	0.757±0.021^aAB^	0.360±0.010^aA^	173±2.1^aE^	10.00±0.50^bC^	0.217±0.021^aBC^
		3	1277±23.1^aAB^	0.727±0.025^aA^	0.380±0.010^aA^	193±1.2^aEF^	12.10±0.32^aD^	0.223±0.025^aC^

9% CS	raw	0	309±27.3^bABC^	0.853±0.025^aA^	0.403±0.032^aABC^	183±1.5^aDE^	11.00±0.20^bB^	0.160±0.010^aAB^
		3	1072±24.7^aD^	0.830±0.020^aA^	0.420±0.020^aABCD^	210±3.5^aDE^	12.00±0.15^aB^	0.170±0.010^aBC^
	cooked	0	419±21.2^bA^	0.757±0.021^aAB^	0.360±0.010^aA^	173±2.1^aE^	10.60±0.31^bC^	0.217±0.021^aBC^
		3	1113±49.3^aC^	0.737±0.015^aA^	0.380±0.010^aA^	187±2.3^aEF^	13.90±0.10^aB^	0.223±0.025^aC^

12% CS	raw	0	269±20.9^bC^	0.853±0.025^aA^	0.353±0.021^aC^	150±1.0^aF^	12.10±0.21^bA^	0.190±0.010^aA^
		3	950±78.1^aE^	0.830±0.020^aA^	0.370±0.020^aD^	170±1.0^aF^	12.80±0.27^aA^	0.203±0.015^aA^
	cooked	0	408±19.0^bA^	0.767±0.021^aA^	0.313±0.015^bB^	133±1.5^bF^	12.10±0.32^bB^	0.247±0.025^aAB^
		3	1033±76.4^aC^	0.737±0.015^aA^	0.357±0.021^aAB^	167±1.5^aF^	15.20±0.25^aA^	0.267±0.015^aAB^

Mean values (±SD); means followed by different capital letters in the same column (effect of treatments) are significant by Duncan's multiple test (*p* ≤ 0.05). Means followed by different small letters in the same column (effect of storage time) are significant by Duncan's multiple test (*p* ≤ 0.05).

## Data Availability

The datasets generated during and/or analyzed during the current study are available from the corresponding author on reasonable request.
